# The Prevalence of Hearing Loss in Patients with Hepatitis B Infection Compared with Healthy Volunteers

**Published:** 2017-05

**Authors:** Elaheh Gholami Parizad, Houshang Gerami Matin, Eskandar Gholami Parizad, Afra Khosravi

**Affiliations:** 1 *Clinical Microbiology Research Centre, Ilam University of Medical Sciences, Ilam, Iran.*; 2 *Nose and Sinus Disease Research Canter, Amiralmomenin Hospital, Guilan University of Medical Science, Rasht, Iran.*; 3 *Psychosocial Injuries Prevention Research Centre, Ilam University of Medical Sciences, Ilam, Iran.*

**Keywords:** Audiometry, Hepatitis B infection, Hearing loss, Healthy volunteers

## Abstract

**Introduction:**

Hepatitis B virus is a virus that creates significant hepatic and extra-hepatic complications, with widespread prevalence across the community and body systemic involvement, and can impact on hearing performance. This study aims to evaluate hearing loss among individuals with hepatitis B compared with healthy subjects.

**Materials and Methods:**

In this case-control study, 83 HBsAg-positive patients with a 1-year history of disease were selected for pure tone audiometry (PTA) testing, while 108 HBsAg-negative patients were selected as the control group. Subjects in both groups were aged 20–40 years. The threshold was set at 25 db for hearing loss. Final data were analyzed using SPSS software.

**Results:**

Significant differences were found between the case group and control group in average PTA and hearing loss. There was also a significant difference between the two groups in average PTA at frequencies of 250, 4,000, and 8,000 Hz, but not at speech frequencies of 500, 1,000 and 2,000 Hz, despite the difference in average PTA.

**Conclusion:**

According to significant differences in average PTA between patients with hepatitis B virus and healthy subjects in this study, hearing loss may be attributed to the presence HBV of in the patient group.

## Introduction

Hepatitis B virus (HBV) is one of the most prevalent endemic infectious agents throughout the world (1). The importance of the disease is due to its high prevalence and hepatic/extra-hepatic complications. Today, three-quarters of the world's population live in *areas* with a *high prevalence of* infection (1–3).

*Acute complications of hepatitis B* range from fulminant hepatitis to manifestations such as hepatic fibrosis, liver *cirrhosis *and hepatocellular carcinoma, with approximately 1 million people a year dying from such complications (1,2). Over the prolonged course of the disease (several years), usually occurring in virus carriers or subjects with hidden hepatitis B, the virus can integrate its DNA into the host DNA and use the host replication system to reproduce its genome. Consequently, various complications arise such as development of hepatocellular carcinoma (HCC) (2). Approximately 5% of individuals with hepatitis B are virus carriers without serious clinical symptoms (3). The presence of the virus has been proven in most body secretions involving blood and its products, sweat, urine, saliva, sexual secretions, breast milk, and cerumen (4).

Several factors impact on sensorineural hearing loss including genetic background, age, use of some antiviral drugs and antibiotics, acoustic trauma, physical trauma, metabolic dysfunctions such as thyroid hormone disease, immune system disorders such as kidney and autoimmunity diseases, and diabetes (5–7). In addition, bacterial infections such as *meningitis caused by* *Streptococcus pneumonia* affects the hearing of 10% of patients, particularly in children in the early course of the disease (8). Specific viral agents including infections caused by mumps, measles, rubella, influenza, and cytomegalovirus can directly affect hearing loss, with the impact being either unilateral or bilateral, and ranging from minor to severe hearing loss and even permanent deafness (9). Viruses causing congenital hearing loss are cytomegalovirus, rubella, and *lymphocytic choriomeningitis virus* (LCMV) (9,10). The viruses that create acquired hearing loss include measles, varicella zoster virus, mumps, and West Nile virus. The viruses that cause congenital and acquired hearing loss are human immunodeficiency virus (HIV) and herpes simplex virus types 1 and 2 (9,10). Parvovirus and B19 viruses can also be responsible for autoimmune inner ear disease (11). The relationship between HBV and *necrotizing vasculitis and polyarthritis nodosa* (PAN), diseases that involve the arteries, veins, and capillaries and can lead to impaired hearing, is quite clear. In fact, 30% of people with PAN are hepatitis B carriers, and are more susceptible to HBV infection (12–14). According to the results obtained from a number of studies, HBV can affect quantitative and qualitative hearing performance, likely due to the importance of hearing in daily activities, quality of life, the high prevalence of this virus in the community, and the involvement of different body systems by the virus.

## Materials and Methods

This case-control study was conducted at the Nourooz-abad Polyclinic and Reference Lab from March 2012 to September 2013 in the Iranian city of Ilam (*located in the west of Iran*). The project was approved by the Medical Ethical Committee of the University of Medical Science, Ilam, Iran (Ir. medilam. rec.1394.90).

A total of 95 HBsAg-positive patients with medical records in the center of reference were selected for this study, as well as 150 healthy, HBsAg- and HbeAg-negative volunteers who acted as the control group. All participants were informed about the project and provide written informed consent. Inclusion criteria were patients with a 1-year history of disease and a positive HBsAg test (Diaplus-Qiagene Elisa Kit, USA). 

The control group were required to have no history of hepatitis B and a negative HBsAg test. Other inclusion criteria for both groups were age 40 years or younger and a lack of history of physical trauma, autoimmune disease, ear infection, tumor, ear trauma, taking antiviral drugs, measles, mumps, rubella, polyarthritis nodosa, work experience in a noisy environment, and a history of hearing loss or deafness in the family. Participants were also required to have no abnormalities in the ear canal following *otoscope review*. After the second screening, 83 and 108 participants were included in the case and control groups, respectively. All participants in both groups were aged between 20 and 40 years. Based on initial examination by otoscopy, no defects in the tympanic or ear infections were observed in either group. Hearing assessment was carried out by an expert using an audiometric devise (OSCILLA SM 96 USA). PTA, and pure tone average for left ear (PTALE) and right ear (PTARE) were performed at frequencies of 250, 500, 1,000, 2,000, 4,000, 8,000 Hz. In addition, speech discrimination score (SDS) at frequencies of 500 to 2,000 and hearing loss percentage (HLP) were estimated. Data were analyzed using SPSS 16.1 and the Kolmogorov-Smirnov test was used to assess the normality of the data. Mann-Whitney and Kruskal-Wallis tests were performed. Hearing loss in this study was classified into five grades according to the American National Standards Institute and American Academy of Otolaryngology. The threshold was set at 25 db for hearing loss.

## Results

In this study, 83 HBsAg-positive patients with mean ± standard deviation (SD) age of 33 ± 6.8 years were selected. The sample *consisted of 62*% *males and 38*% *females*. One hundred and eight HBsAg-negative subjects were included in the control group, consisting of 27% females and 73% males, with a mean±SD age of 28±5.5 years. *In the investigation of relationship* among the average PTA, degrees of hearing loss at frequencies of 250 to 8,000 Hz and age and sex, no significant differences were observed in the control group. There was a significant correlation between left and right PTA with age in the patient group; this difference was observed in the age group 30–40 years (P=0.02). PTA for each frequency in both ears in the case and control groups is presented in (Table.1).

**Table 1:** Comparison of pure tone audiometry (PTA) at different frequencies in case and control groups

**Frequency** **in ear (Hz**) **Group**	**250**		**500**		**1,000**		**2,000**		**4,000**		**8,000**	
	R	L	R	L	R	L	R	L	R	L	R	L
Patients (n=83)	22.2	20.2	17.1	16.6	12.7	9.6	15.4	12.3	30.5	27.3	22.5	21.1
Volunteer (n=108)	16.9	15.3	13.9	12.8	12.6	11.1	14.2	12	21.5	18.7	14.7	12.2
P-value	0.027*	0.023*	0.176	0.06	0.73	0.17	0.57	0.12	0.001*	0.00*	0.00*	0.004*

* statistically significant

According to Table 1, there were significant differences in PTA at frequencies of 250, 4,000, 8,000 Hz between the case and control groups. However, average PTA in both ears was higher than the 25-db threshold in the case group at 4,000 Hz only (Table.2). Average PTARE was 20.03 db and average PTALE was 18.22 db from 250 to 8,000 Hz in patients (Table.2). In the control group, the average PTA at the six frequencies in PTARE was 15.73 db and 13.76 db in PTALE (Table.2). 

**Table 2:** Pure tone average (PTA), hearing loss percentage (HLP) and speech discrimination score (SDS) at different frequencies in ears of both case and control groups

**Variable Group**	**PTARE**	**PTALE**	**HLPRE**	**HLPLE**	**SDSR**	**SDSL**
Patients (n=83)	20.03	18.22	5.10	4.04	14.96	13.17
Volunteer (n=108)	15.73	13.76	1.78	1.09	13.49	11.95
P-value	0.018*	0.005*	0.037*	0.014*	0.431	0.467

* statistically significant SDSR: Speech Discrimination Score in Right Ear, SDSL: Speech Discrimination Score in Left Ear, PTA: Pure Tone Audiometric, ABR: Auditory Brain Stems Response, PTARE: Pure Tone Average in Right Ear, PTALE: Pure Tone Average in Left Ear, HLPLE: Hearing Loss Percentage Left Ear, HLPRE: Hearing Loss Percentage Right Ear

A statistically significant difference was observed in total average PTALE and PTARE in both groups (Table.2). The percentage of hearing loss in the right and left ear (HLPRE and HLPLE) over the total of six frequencies differed significantly in the two groups (Table.2), although not at frequencies of speech of 500, 1,000 or 2000 Hz (Table.2). The highest average percentage of hearing loss at frequencies of speech of 500, 1,000, and 2,000 Hz was in the first grade (26–40) of hearing loss in the right ear in the case group and in the second grade of hearing loss in the control group. However, there was no significant difference between degrees of hearing loss and frequencies of speech discrimination in both groups (Table.3).

**Table 3:** Hearing loss percentage (HLP) at frequencies of speech discrimination score (SDS) according to degrees of hearing loss in case and control groups

**Frequency of SDS (Hz)**	**500**				**1000**				**2000**			
Hearing loss percentage in both ear and groups *Grade	Patients (%)		Volunteers (%)		Patients (%)		Volunteers (%)		Patients (%)		Volunteers (%)	
	R	L	R	L	R	L	R	L	R	L	R	L
1	3.7	3.7	8.3	1.9	3.7	1.2	5.6	2.8	6.1	6	7.4	5.6
2	1.2	2.4	0	0.9	3.7	1.2	1.9	0.9	4.6	2.4	1.9	2.8
3	0	1.2	0	0	0	1.2	0.9	0.9	2.4	2.4	2.8	1.8
4	0	0	0	0	0	0	0	0	0	0	0	0
5	0	0	0	0	0	0	0	0	0	0	0	0

**Grade 0** 0–25 No Impairment **Grade 1** 26–40 Slight Impairment **Grade 2** 41–55 Moderate Impairment

**Grade 3** 56–70 Severe Impairment **Grade 4** 71–90 Profound Impairment **Grade 5**>90Permanent Deafness

* In this table according to the American National Standards Institute and American Academy of Otolaryngology (based on frequencies of 500, 1000, and 2000 Hz) the percentage of patients and volunteers with different degree of hearing loss distributed in five grade divisions:

## Discussion

Many factors contribute to hearing loss, and almost 10% of people experience hearing loss ranging from mild to severe for various reasons (6). Hearing loss patterns may be observed in any way and refer to sensorineural hearing loss when the inner ear or auditory nerve are damaged. This type of hearing loss is generally stable and is not treatable with medication, and affects the quality of the sense and consequently quality of life (6,7). The causes of sensorineural hearing loss are not clearly identified, but the development of some viral diseases is known to be one important factor. For example, it has been proven that cytomegalovirus (CMV) causes severe sensorineural hearing loss in 6–6.5% of infected people, while rubella and LCMV cause moderate-to-severe sensorineural hearing loss in 12–19% and 7.4% of affected subjects, respectively (5,9). Other viruses such as HIV (33.5–27.5%),HSV (over 33%), varicella zoster virus (VZV) (7–85%), measles (0.1–3.4%), and mumps (0.005–4%) cause sensorineural hearing loss in different proportions of patients (5,9,15). Furthermore, studies have found that infections that produce anti-phospholipids antibodies (APLA) such as HIV, Epstein-Barr virus, parvovirus and hepatitis A, B and C viruses, mumps, rubella and syphilis are associated with hearing loss in patients with autoimmune diseases (16).

The presence of HBV in the cerumen has been demonstrated in patients infected by hepatitis B (5), and also the relationship between the risk of this type of infection with certain autoimmune diseases such as polyarthritis is important in terms of hearing loss (10,12,13). Thus, the presence of HBsAg, or immunoglobulin complex against HbsAg, in the blood of people who already have experienced the disease can be detected in 30% of patients with PAN (12). In fact, hepatitis B is caused by PAN and can disrupt immune system function and accordingly hearing loss (12,10). Furthermore, in a 2009 case report by Huang et al., it was suggested that unilateral hearing loss is likely by exacerbation of hepatitis B, an increase of virus copies in the serum (17,18).

According to the findings of the present study, the average PTA in both ears of patients aged 30–40 years showed significant differences, which might suggest that the virus has a greater impact on age-related hearing loss in people with hepatitis B infection and prolongs the duration of chronic disease compared with healthy people. However, more studies are needed to prove this hypothesis.

By comparing the PTA for each frequency, significant differences can be observed between the two groups at frequencies of 250, 4,000, and 8,000 Hz. Average PTA was greater than the 25-db threshold of hearing loss in patients only at a frequency of 4,000 Hz in both ears (Table.1). In a study conducted by Shayaninasab et al. in 2012 on 97 patients and 97 controls, the highest rate of hearing loss occurred at frequencies between 500 and 8,000 Hz (14). The difference between these two studies may be due to the different thresholds used for hearing loss (20 db in the Shayaninasab study compared with 25 db in the present study). In addition, the average PTA in the case group for both left and right ears were consistent with previous studies, while this average in the control group in present study was greater than in other studies (but less than 25 db; Table.2). Also, given that none of the patients in the present study had any history of PAN or HCV diseases and had not received any vaccines or antiviral drugs, minor-to-moderate hearing losses were observed at some frequencies. However, the relationship between hearing loss and the presence of this virus was not significant among patients at some frequencies (Table.3).

 Furthermore, no significant differences in speech discrimination were shown for the two cases and the control groups, similar to previous studies. The highest percentage of hearing loss in the case group was observed in the first grade (26 to 40 decibels), which was less than the control group. Noting that the frequencies of speech include core understanding of speech in daily conversation, any disruption in these frequencies will have a large effect on hearing quality (14, 19, 20). However, considering that a greater percentage decrease was observed in the control group, the reason may be found in factors other than HBV infection and other viral diseases. However, the number of people with hepatitis B and moderate hearing loss in the second grade (41–55 db) was significantly greater than that in the control group in this grade. It should be noted that there was no significant difference among various degrees of hearing loss and frequencies of speech in both groups. In addition, severe hearing loss (70–90 db) and unilateral or bilateral permanent deafness (<90 db) were not observed in either of the groups, which is consistent with previous studies (14).

## Conclusion

The findings revealed that there is a relationship between hearing loss and HBV in patients with HBV. Access to a wider statistical population, medical history, and family diseases and investigation of auditory brainstem response (ABR) in future studies may help to clarify the role of hepatitis B in causing hearing loss in individuals infected by HBV, as well as the study of liver enzyme factors and other specific tests. Considering the few studies conducted in this area, hearing loss in some patients with hepatitis B may be attributable to the presence of the virus in the body; although further studies are required.
